# Detection of *Legionella *by quantitative-polymerase chain reaction (qPCR) for monitoring and risk assessment

**DOI:** 10.1186/1471-2180-11-254

**Published:** 2011-11-21

**Authors:** Louise H Krøjgaard, Karen A Krogfelt, Hans-Jørgen Albrechtsen, Søren A Uldum

**Affiliations:** 1 Dept. of Microbiological Surveillance and Research, Statens Serum Institut, Ørestads Boulevard 5, 2300 Copenhagen S, Denmark; 2 Dept. of Environmental Engineering, Technical University of Denmark, Miljoevej bygning 113, 2800 Kgs. Lyngby, Denmark

## Abstract

**Background:**

Culture and quantitative polymerase chain reaction (qPCR) assays for the detection of *Legionella *were compared on samples from a residential area before and after two interventions. A total of 84 samples were collected from shower hoses and taps as first flush samples and at constant temperature. Samples were grouped according to the origin of the sample, a) circulation water b) water from empty apartments c) water from shower hoses. The aims were to investigate the usefulness of qPCR compared to culture for monitoring remedial actions for elimination of *Legionella *bacteria and as a tool for risk assessment.

**Results:**

In water collected from the apartments *Legionella *spp were detected by qPCR in the concentration range from LOQ to 9.6*10^5^GU/L while *L. pneumophila *were detected in a range from LOQ to 6.8*10^5 ^GU/L. By culturing, the legionellae were detected in the range from below detection limit (> 10 CFU/L) to 1.6*10^6 ^CFU/L. In circulating water and in first flush water from shower hoses, culture and qPCR showed the same tendencies. The overall correlation between the bacteria number detected by culture and the two developed qPCR assays (*L*. spp and *L. pneumophila*) was relatively poor (r^2 ^= 0.31 for culture and *Legionella *spp. assay, r^2 ^= 0.20 for culture and *L. pneumophila *assay).

**Conclusion:**

Detection by qPCR was suitable for monitoring changes in the concentration of *Legionella *but the precise determination of bacteria is difficult. Risk assessment by qPCR only on samples without any background information regarding treatment, timing, etc is dubious. However, the rapid detection by qPCR of high concentrations of *Legionella *- especially *Legionella pneumophila *- is valuable as an indicator of risk, although it may be false positive compared to culture results. On the other hand, the detection of a low number of bacteria by qPCR is a strong indication for the absence of risk.

## Background

*Legionella *bacteria are ubiquitous in nature and are often found in natural water sources as well as in man-made water systems. Humans may be infected through inhalation of contaminated aerosolised water droplets. Symptoms range from influenza-like disease (Pontiac fever) to severe pneumonia (Legionnaires' disease, LD) with a high mortality rate [1,2]. More than 50 *Legionella *species are described, but *Legionella (L.) pneumophila *is the cause of more than 95% of LD cases [3,4]. High concentrations (10^4^-10^10 ^*Legionella *CFU/L) of *Legionella *in the water sources are considered a risk of infection [5-8]. Being able to determine the concentration of *Legionella *in water is, therefore, highly relevant in risk assessments and transmission tracing. The reference method for enumeration of *Legionella *in water is culture [9]. Culture is, however, hampered by a long incubation time (7 to 10 days) whereas qPCR can be performed within three hours. By culture, only bacteria cultivable under the given conditions can be quantified in environmental samples with mixed cultures of different bacteria including different *Legionella *species. Quantitative detection of *L. pneumophila *(which is the most significant *Legionella *species for risk assessment) is difficult by culture. In qPCR, with specific *L. pneumophila *primers, only *L. pneumophila *will be amplified irrespectively of background flora etc. The important bias of qPCR compared to culture is that also dead and otherwise not culturable *Legionella *will be quantified.

The aim of this work was to clarify under which circumstances and in which samples qPCR is useful for monitoring and risk assessment. The investigation was performed in a newly built residential area where two males contracted LD.

## Methods

### The sampling area and the interventions

A newly built residential area associated with two cases was investigated [10]. The area consisted of 225 apartments distributed on 6 blocks; around 210 apartments were inhabited at the time of the sampling period. The two cases and the interventions done to overcome the *Legionella *colonisation and the risk factors found to be associated with the residential area were published previously [10].

Two interventions were conducted to control the *Legionella *contamination of the hot water system. The first was a 12 h heat treatment of the boilers (approximately 70°C) together with a request to all residents to flush their taps for 5 minutes. Subsequently, the water in the boilers was completely replaced with fresh water and the temperature was lowered to 60°C. Circulation pumps were set at maximum flow.

As the first intervention did not reduce the *Legionella *count satisfactorily, a second intervention was performed three weeks later, which consisted of an increase of the water temperature in the boilers to approximately 70°C for 24 hours. During this time, all taps were flushed for 5 min. The boilers were hyperchlorinated and the temperature was set at 65°C. All shower hoses were replaced with new ones in all apartments, and over the next month the boiler temperature was regulated to ensure that the water in the most distant taps was kept at > 50°C. Taps of empty apartments were flushed weekly for 5 min with water from the hot water taps.

### Sampling and analysis

Water samples (a total of 84) were collected at ten occasions from January 9, 2009 to September 7, 2009: One sampling before any of the interventions, three samplings between the first and the second interventions and six samplings after the second intervention.

Water samples included the first one litre of water from the tap (first flush, A samples Table 1) and one litre collected after flushing until constant hot water temperature was obtained (B samples Table 1). Samples were collected from kitchen and bathroom taps as well as from shower hoses.

**Table 1:** Comparison of culture and qPCR for quantification of *Legionella*.

				Number of positive samples			Concentrations		
				
				Culture	qPCR		Culture	qPCR	
				
Sampling	Sampling site	Type of sample	No of samples	*Legionella *spp	*Legionella *spp	*Legionella pneumophila *	*Legionella *spp 10^4 ^CFU/L	*Legionella *spp10^4 ^GU/L	*Legionella pneumophila *10^4 ^GU/L
	Circulation water	B	1	1	1	1	5.5	3.4	3.6
	
Before the first intervention	Empty apartment	A	0						
	
	Shower hose	A	1	1	1	1	60	26	14

	Circulation water	B	10	10	10	10	0.005 - 1.2 [0.08]	0.77 - 2.9 [1.5]	0.6 - 2.6 [1.1]
	
After the first intervention	Empty apartment	A	4	4	4	4	1.9 - 33 [19]	2.9 - 24 [8.9]	4.9 - 19 [11]
	
	Shower hose	A	5	5	5	5	0.8 - 160 [27]	3.5 - 96 [28]	1.1 - 43 [17]

	Circulation water	B	16	0	16	13	BD	0.4-1.9 [0.62]	BD - 2.0 [0.27]
	
After the second intervention	Empty apartment	A	2	1	2	2	BD - 0.001	3.2 - 55 [29]	3.7 - 68 [36]
	
	Shower hose	A	8	0	8	8	BD	0.17 - 2.3 [0.95]	0.033 - 3.2 [1.3]

Number of samples and amount of *Legionella *detected in samples from circulation water, from first flush of taps in empty apartments and from first flush of shower hoses by culture and by *Legionella pneumophila *and *Legionella *species qPCR assays before and after interventions. Samples were collected as first flush (A) or after reaching constant temperature (B). BD: Below detection. Median value is given in [..]

### Culture and extraction of DNA for qPCR

Culture procedure followed the ISO standard 11731-2: 2006 on both MWY (Modified Wadowsky Yee) (Oxoid, Greve, Denmark) and GVPC (Glycine, Vancomycin, polymyxin, Cycloheximide) (Oxoid, Greve, Denmark) agar plates and based on three different concentration steps. DNA extraction was performed from a 100 fold concentration of the water samples, with Chelex^®^100 (Bio-Rad California, USA) (900 μL sample, 150 μL Chelex^®^100) before qPCR. Culturing of samples was previously described in detail in Krøjgaard *et al *(2011) [10]

### qPCR *Legionella *species and *Legionella pneumophila *assay

qPCR was performed with primers and a probe detecting *Legionella *species (targeting the *5S *rRNA gene) and primers and a probe detecting *L. pneumophila *(the *mi*p gene); both primers and probes were optimized to a TaqMan assay. Internal process controls (IPCs) for *Legionella *spp. and *L. pneumophila *were included in order to assess inhibition or suboptimal reaction conditions. The IPC was co-amplified in every qPCR reaction together with target DNA [11].

Standard dilution was correlated to the French *Legionella pneumophila *DNA standard (SMR_ LEGDNA_01 standard from Legionelles centre National de Référence, Lyon, France). The limit of detection (LOD) was 2.5 GU/reaction (5 μL) for the *Legionella *species assay corresponding to 833 GU/L. The limit of quantification (LOQ) was 10 GU/5 μL and 3333 GU/L. The LOD for the *L. pneumophila *assay was 15 GU/5 μL / 5000 GU/L and the LOQ was 25 GU/5 μL/8333 GU/L.

## Results & discussion

### Overall correlation between qPCR and culture

*Legionella *species were detected by qPCR in all 84 water samples. Four samples were below LOD. *L. pneumophila *were detected in 75 of the 84 samples, in 34 samples below LOQ. Forty-tree of the 84 samples were found positive by culture. The amount found by culture and qPCR for all (84 samples) did not correlate well (r^2 ^= 0.31 *L*. species assay, r^2 ^= 0.20 *L. pneumophila *assay). Poor correlations were also found in others investigations comparing culture and qPCR results for samples from hot water systems [12-15]. qPCR amplifies DNA from both living and dead *Legionella *still harbouring the DNA. An effective heat treatment would, therefore, not necessarily significantly change the amount detected by qPCR in the short term, as long as the cells not had lost their DNA. When *Legionella *DNA is still in the water system, it can be amplified by qPCR. By culture depending on living and culturable bacteria, no or only limited growth would be expected after effective heat treatment. This effect of temperature is supported by Lee *et al *(2011) [16] who found a significantly higher mean log difference comparing culture and qPCR at temperatures above 50°C than at lower temperatures. Comparison of the methods for samples collected from water systems where no interventions have been conducted, would probably give a better agreement between the two methods.

### Circulation water

**Figure 1:**
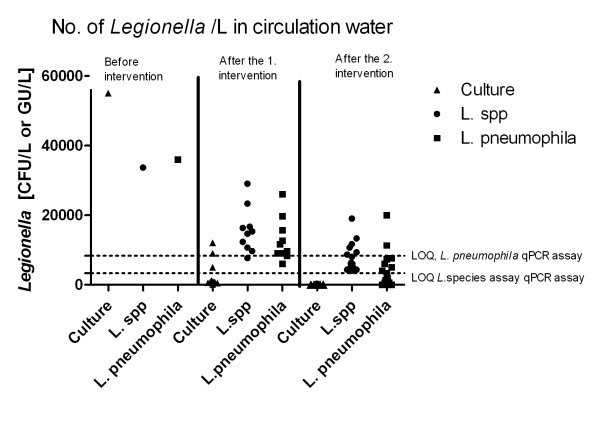
**Circulation water**. Comparison of the amount of *Legionella *detected by culture and by qPCR. *Legionella *species and the *Legionella pneumophila *assay in circulation water before and after the two interventions. LOQ: Limit of quantification. Water samples were collected from both bathroom and kitchen hot water taps. Each triangle, dot and square represents one sample.

To investigate under which circumstances qPCR could be applicable for monitoring and risk assessment, the samples were grouped according to collection history. The amount of *Legionella *found in circulation water (water with constant temperature) before and after the two interventions showed the same tendency both by culture and qPCR (Figure 1 and Table 1). The amount of *Legionella *detected by both methods (and both primer assays) decreased after each treatment. Before any treatment, 5.5*10^4 ^*Legionella *CFU/L was found by culture, 3.4*10^4 ^GU *L*. species/L and 3.6*10^4 ^GU *L. pneumophila */L was found by qPCR. The discrepancy between the amounts found by culture and qPCR is probably due to loss of bacteria in the concentration steps conducted before qPCR. Culture is based on the amount found also in unconcentrated samples with no loss.

After the first intervention, *Legionella *was found in lower concentrations (< 10*10^3 ^*Legionella *CFU/L) in six out of ten samples. The four other samples were in the range 1.2*10^3^ - 1.2*10^4 ^*Legionella *CFU/L. The range measured by qPCR after the first intervention (both assays) was from 6.0*10^3 ^to 2.9*10^4 ^GU/L (Table 1).

After the second intervention, no legionellae were detected by culture, but the range found by qPCR for the *L*. species assay was 4.0*10^3 ^to 1.9*10^4 ^GU/L with an median of 6.2*10^3^ GU/L. For the *L. pneumophila *assay, three samples were negative, but the 13 other samples were positive ranging from 6.7*10^2 ^to 2.0*10^4 ^ GU/L (Table 1).

The second intervention seemed to kill or make *Legionella *uncultivable but the results from qPCR showed that they were still present in the system as dead or uncultivable bacteria. There was no obvious difference between the amount detected just after the second intervention and seven months after measuring *Legionella *species. By qPCR, the amount of *L. pneumophila *was found to decrease slightly with time. The ranges in which *Legionella *were detected before and after the second intervention measured by qPCR on circulation water samples were overlapping. Therefore, it is difficult to draw conclusions on the effect of the remedial actions and to form a picture of the risk using the distinct values from circulation water provided by qPCR; however, trends or tendencies can be detected.

### First flush from empty apartments

**Figure 2:**
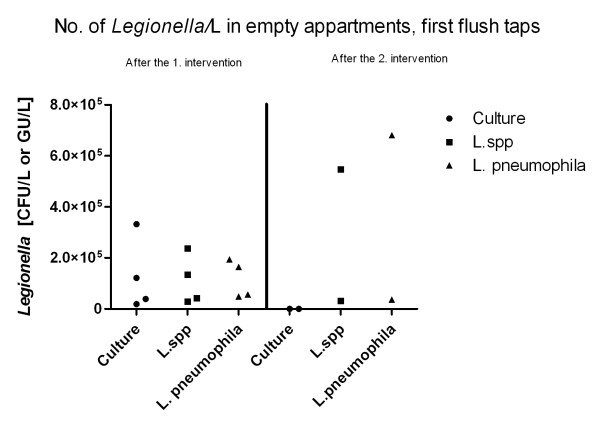
**Empty apartments first flush**. Comparison of the amount of *Legionella *detected by culture and by qPCR. *Legionella *species and the *Legionella pneumophila *assay in first flush samples from empty apartments before and after the second intervention. Collected from both bathroom and kitchen hot water taps. Each triangle, dot and square represents one sample

Stagnancy of water at an ambient temperature induces an increased risk of *Legionella *growth. In building blocks, the pipelines leading to each apartment could constitute local areas with stagnant water if an apartment is left unoccupied, which could lead to colonisation of the whole water system. To minimise this risk, a procedure of flushing with hot water (> 50°C - 70°C) of taps for 5 min each was introduced (part of intervention II) for empty apartments. To measure the effect of the remedial measures and to assess the risk associated with stagnant water, first flush samples from empty apartments were analysed by qPCR and culture (Figure 2). Since no samples were collected before the interventions, we only have data before and after the second intervention. Before the second intervention, the amount found by culture and qPCR were generally equal. Samples contained from 1.9*10^4 ^to 3.3*10^5 ^*Legionella *CFU/L (culture), and 2.9*10^4 ^to 2.4*10^5 ^GU/L (*L*. species) and 4.9*10^4 ^to 1.9*10^5 ^GU/L (*L. pneumophila*) (qPCR) as shown in Table 1. After the second intervention, 10 CFU/L and no *Legionella *CFU/L, respectively, were found by culture in two samples (same apartment at a six-month interval). The one sample of these two samples showed by qPCR 5.5*10^5 ^GU/L (*L*. species) and 6.8*10^5 ^GU/L (*L. pneumophila*) and the other sample showed 3.2*10^4 ^GU/L (*L*. species) and 3.7*10^4 ^GU/L (*L. pneumophila*) (Table 1).

Regular flushing seemed to control the level of live *Legionella *in the distant parts of the pipes in the empty apartments over a long period, but this could not be demonstrated with qPCR. Sudden opening of the tap could probably flush out biofilm with dead *Legionella *which could be detected by qPCR but not by culture. It can be concluded that qPCR could not be used for risk assessment or for monitoring the effect of the remedial actions on stagnant water in an empty apartment. It should be noted that only water from one apartment was sampled after the second intervention.

### First flush from shower hoses

**Figure 3:**
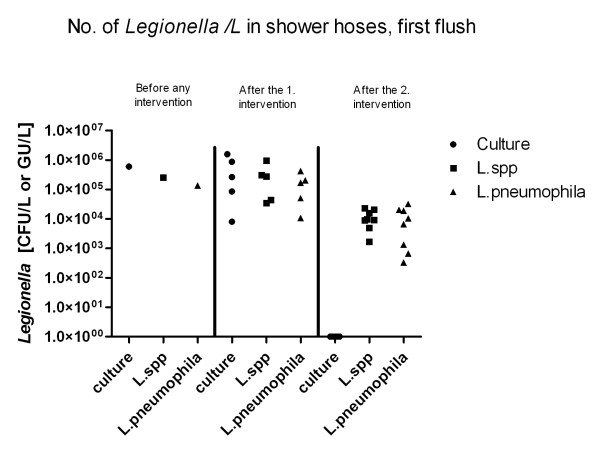
**Shower hoses first flush**. Comparison of the amount of *Legionella *detected by culture and by qPCR. *Legionella *species and the *Legionella pneumophila *assay in first flush samples from shower hoses before and after the two interventions. LOQ: Limit of quantification. Each triangle, dot and square represents one sample

Infection with *Legionella *is caused by inhalation of aerosolised contaminated water droplets and both shower heads and shower hoses provide an environment for high *Legionella *concentrations [17]. One major route of infection could be contaminated water from showers. The first litre of water from the shower hose and from the end of the pipe system was collected. Before any interventions were initiated, the first flush collected from one shower hose contained almost the same amount of legionellae, irrespective of the methods used (6.0*10^5 ^*Legionella *CFU/L, 2.6*10^5 ^GU/L *L*. species and 1.4*10^5 ^GU/L *L. pneumophila */L) (Figure 3). After the first intervention, the range of *Legionella *found with each of the methods and each of the qPCR assays, were both below and above the level found before the intervention (one single apartment). After the second intervention, seven out of eight samples showed no growth of *Legionella *by culture (this was after the replacement of the shower hoses). The one positive sample contained only 50 *Legionella *CFU/L. Measuring the same eight samples by qPCR, the level had decreased (range 1.7*10^3 ^- 2.3*10^4 ^GU/L *L*. species, median 9.5*10^3^, range 3.3*10^2 ^- 3.2*10^4 ^GU/L *L. pneumophila*, median 1.3*10^4 ^(Table 1).

The conclusion for samples from shower hoses is the same as for circulation water. qPCR is suitable for monitoring a change in the bacterial concentration, whereas the specific number of bacteria is difficult to use for risk assessment.

Overall, when using qPCR, background information on the system from where samples have been collected is helpful in the interpretation of the results. Knowledge of any treatments of the water and the temperature profile of the system is essential for the interpretation. If water is collected at a temperature known to support the growth of *Legionella*, and no disinfection has been conducted, the high amount of *Legionella *found by qPCR is also found by culture. If disinfection of some kind was used it is more difficult to correlate results from the two methods since some or all *Legionella *could have been killed. However, on some occasions it could be interesting to monitor the level of dead or unculturable *Legionella*, since a high level measured by qPCR could indicate a current or recent colonisation of the system, which could indicate a potential risk even though the bacteria do not grow.

As also discussed in Joly *et al *2006 [14] a negative or low level of *Legionella *detected by qPCR is a quite good predictor of a negative culture result. Unfortunately, this selection is difficult to establish based on detection of *Legionella *species since all tested samples were found to contain *Legionella *DNA. Using the *Legionella pneumophila *assay, eight of ten samples found negative by qPCR were also negative by culture.

It has been suggested to improve the usefulness of qPCR by pre-treatment with the DNA-dye Propidium monoazide to discriminate between dead and live bacteria [18]. Previous work with dying DNA of membrane compromised cells focused on the use of the dye ethidium monoazide [19] but Propidium monoazide has been found to show less cytotoxicity [18]. Nevertheless, optimization of the use of the dyes is still needed.

## Conclusion

We found that detection of *Legionella *in water samples by qPCR was suitable for monitoring changes in the concentration of *Legionella *over time, whereas the specific number measured by qPCR was difficult to use for risk assessment. Results for both culture and qPCR followed the same decreasing tendencies for circulating water and first flush water samples from shower hoses. In first flush samples from empty apartments, before the second intervention, culture and qPCR results were generally at the same level, but the two samples collected after the second intervention showed different tendencies with the two methods.

Background information about the water system is necessary to interpret the qPCR results, but low amounts of *Legionella pneumophila *detected by qPCR is a good indicator of low risk, and detection of high levels in untreated water systems is a good indicator of colonisation and risk.

## Competing interests

The authors declare that they have no competing interests.

## Authors' contributions

LHK: Was involved in the decision making on choosing the locality of apartments and tap locations. Collected most samples. Helped during concentration and cultivation of samples. Purified DNA from samples and tested them on qPCR. Had the main responsibility and workload of all data analyses. Was active in the interpretation of the results. Wrote the article. Has read and approved the final manuscript. SU: Has participated actively in all parts of the process. Responsible for the diagnostics of patients and environmental isolates together with unravelling the source of infection. Responsible for the culture analysis of water samples. Involved in decisions about choosing the locality of apartments and tap locations. Involved in preparation of the manuscript. Has read and approved the final manuscript. KAK: Contributed to designing the study, involved in discussing the results and building the article. Has read and approved the final manuscript. HANA: Contributed to planning, judging and interpretation of the results and building the article. Has read and approved the final manuscript.
